# Safety in psychiatric inpatient care: The impact of risk management culture on mental health nursing practice

**DOI:** 10.1111/nin.12199

**Published:** 2017-04-18

**Authors:** Allie Slemon, Emily Jenkins, Vicky Bungay

**Affiliations:** ^1^ School of Nursing University of British Columbia (UBC) Vancouver British Columbia Canada

**Keywords:** mental health, nursing practice, patient safety, risk management

## Abstract

The discourse of safety has informed the care of individuals with mental illness through institutionalization and into modern psychiatric nursing practices. Confinement arose from safety: out of both societal stigma and fear for public safety, as well as benevolently paternalistic aims to protect individuals from self‐harm. In this paper, we argue that within current psychiatric inpatient environments, safety is maintained as the predominant value, and risk management is the cornerstone of nursing care. Practices that accord with this value are legitimized and perpetuated through the safety discourse, despite evidence refuting their efficacy, and patient perspectives demonstrating harm. To illustrate this growing concern in mental health nursing care, we provide four exemplars of risk management strategies utilized in psychiatric inpatient settings: close observations, seclusion, door locking and defensive nursing practice. The use of these strategies demonstrates the necessity to shift perspectives on safety and risk in nursing care. We suggest that to re‐centre meaningful support and treatment of clients, nurses should provide individualized, flexible care that incorporates safety measures while also fundamentally re‐evaluating the risk management culture that gives rise to and legitimizes harmful practices.

## 1 Introduction

Across health care environments, the notion of safety invokes a cluster of concepts including patient safety, quality assurance and quality improvement (Hall, Moore, & Barnsteiner, 2008). Safety in nursing practice constitutes protecting patients from harms arising from adverse events in care such as medication errors, poor communication in handover, insufficient staffing or inadequate education on new technologies (Sherwood, 2015). However, within mental health care, discussions of patient safety issues resulting from harms of the health care environment are limited (Kanerva, Lammintakanen, & Kivinen, 2016) and are often replaced by the notion of patient risk: the harms that a patient *creates* within the environment including violence, aggression, self‐harm or suicide (Bowers et al., 2010a; Crowe & Carlyle, 2003; De Santis et al., 2015). In contrast to other hospital environments, within psychiatric inpatient settings, patient risk is conceptualized as affecting not only the individual, but also other patients, staff and the general public, widening the sphere of risk. Lupton (2013) defines risk as the possibility of adverse or dangerous events combined with the belief that prevention of these events is achievable. This paper utilizes Lupton's definition and argues that practices of identifying possible risks and taking preventative action constitute the predominant aim of psychiatric nursing to uphold safety. Nurses uphold safety through adoption of a custodial role with nursing practice (Loukidou, Ioannidi, & Kalokerinou‐Anagnostopoulou, 2010), comprising risk management strategies such as forced medications or the use of seclusion (isolating an individual in a designated locked room) to uphold safety through containment of an individual's behaviour or person (Larsen & Terkelsen, 2014; Muralidharan & Fenton, 2012). Environmental risk management operates towards the same aim and includes locked unit doors, enclosed nursing stations and open “fishbowl” spaces to increase sightlines and facilitate patient observation (Shattell, Andes, & Thomas, 2008). Although some dissenting voices in the mental health field argue that psychological safety, freedom from fear and disempowering experiences, is a key consideration in conceptualizing safety in mental health inpatient environments (Delaney & Johnson, 2008), the discourse of safety is comprised almost entirely of identifying and managing the risks posed by patients during their hospitalization.

In inpatient nursing care within this context, safety is not merely a consideration or goal, but the highest value. As articulated by Bowers, Banda, and Nijman (2010a): “the first purpose of psychiatry is to keep patients and others safe” (p. 315). Mental health researchers and nurses working in psychiatric fields view safety as paramount, and utilize this value to inform nursing interventions, practices and clinical judgement (De Santis et al., 2015; Doyal, Doyal, & Sokol, 2009; Landeweer, Abma, & Widdershoven, 2011; Salzmann‐Erikson, 2015). On the surface, the safety discourse appears congruent with ethical nursing practice, in which risk management implies a moral imperative to protect the patient population, health care providers and the general public through beneficence, prevention of harm and promotion of wellness. However, the dominance of this discourse obscures the often physically and/or psychologically harmful nature of nursing practices designed to uphold safety (Paterson, McIntosh, Wilkinson, McComish, & Smith, 2013; Valenti, Giacco, Katasakou, & Priebe, 2014), undermining the alignment of risk management strategies with ethical practice. Landeweer et al. (2011) argue that the framework of safety in nursing care creates the perception that risk management strategies such as seclusion are necessary, and that they are utilized *only* when necessary. This perception eliminates the place of self‐reflexivity and ethical reflection in nursing care, creating automatic justification for nursing practices. At the individual, institutional and system levels, safety is a well‐intentioned and important value, however, in a context in which patients are frequently detained for involuntary treatment and deemed incompetent to manage risk, safety holds the potential to serve as a carte blanche for nursing practice.

In this paper, we argue that safety, defined as risk identification and associated risk management strategies (Lupton, 2013), is a discourse that gives rise to and legitimizes nursing practices that are ineffective and unethical and eclipse meaningful treatment within psychiatric inpatient settings. We contextualize current perspectives on safety within a history of institutionalization. We then offer four exemplars to demonstrate how the discourse of safety is utilized to inform practices in the management of risks. We conclude with recommendations for reconceptualizing safety and risk within the context of nursing practice and psychiatric inpatient care.

## 2 Safety in the Era of Institutionalization

To understand how the safety discourse became a prominent value in mental health nursing, it is helpful to consider the historical dynamics from which it emerged, including the development of nursing risk management practices. In this section, Goffman's *Asylums* (Goffman 1961) and Foucault's *Madness and Civilization* (Foucault, 1965) are presented as texts that provide a historical context in which stigmatizing societal attitudes and responses to mental illness contributed to the development of institutions designed to contain and keep separate individuals with mental illness from the rest of society. These authors each offer theoretical perspectives that illuminate the legitimization of practices utilized to control risk and uphold safety, and contribute to current understandings of risk management culture in psychiatric nursing practice.

Goffman (1961) argues that society's total institutions (a category that includes jails, concentration camps and mental institutions) remove an individual's connection to the outside world through the development of complex and oppressive internal environments that encompass the individual's entire life. The environment is characterized by surveillance and control, and with admission to a total institution, inmates undergo a mortification in which autonomy and self‐expression are replaced with institutionally mediated behaviours. For example, within the mental institution, inmates are continuously observed and monitored, and are afforded a narrow margin of acceptable behaviour and expression that is not interpreted as symptomatic of mental illness. Individuals who demonstrate behaviour which is deemed disruptive or indicative of disorder face such punitive measures as removal of off‐ground or personal clothing privileges, seclusion in isolation rooms and physical restraint, or bodily harm including starvation and hard labour. Any staff member in the “asylum” may exercise power and control over any inmate, creating an environment in which the restrictions of autonomy are pervasive and unremittent, and mortifications are institutionally sanctioned (Ernst, 2016; Goffman, 1961).

Goffman states that within total institutions, mortifications are “officially rationalized” (p. 46) through an articulated purpose for the existence and operations of the institution: within the mental institution, safety is the rationalization for elimination of freedoms and autonomy of its mentally ill inmates. Goffman describes how the institution's rationalizations give rise to legitimized harmful practices reframed as necessities: “if a suicidal inmate is to be kept alive, the staff may feel it necessary to keep him…tied to a chair in a small locked room” (p. 77). This intervention is not only upholding safety, but serving as treatment itself, providing a further justification for practices. The framing of these interventions as necessities, at its extreme, permits the development of inhumane treatment, such as performing unwarranted hysterectomies and lobotomies to treat mental illness (Beer, 2007; Goffman, 1961). Freedom of action, including movement in the outside world, is reframed as a privilege which must be earned through acceptable and *safe* behaviour. Despite the frequently articulated benevolent intent of asylums, the discourse of safety reinscribes the notion that individuals with mental illness are dangerous, “both incapable of looking after themselves and a threat to the community” (p. 4), and legitimizes the development and maintenance of unethical practices.

While Goffman's work details the process through which the safety discourse provided rationalization for harmful practices in the era of institutionalization, Foucault's *Madness and Civilization* (Foucault, 1965) offers historical context for the development of safety as a rationalization for unethical treatment, illuminating the social forces of fear and stigma that contributed to institutionalization. Foucault argues that throughout history, madness^1^ was constructed through stigma born out of the values of the time: in advance of the current illness discourse, societies have variously considered the mad to be evil, idle and animalistic. Each construct engendered fear of difference and led to the confinement of the mad in jails, and ultimately within mental institutions. Yet while fear created the mental institution, its staff understood and articulated their role as providers of benevolent therapeutic care; this duality of fear and benevolence permitted the twisting of the notion of treatment to consist of the harmful practices described by Goffman. Confinement itself is likewise framed as treatment, with the mental institution termed an asylum: a sanctuary for recuperation and recovery. Society's fear of mental illness encourages the discourse of safety to flourish, with confinement and unethical practices legitimized through their utility in addressing this articulated need for protection.

Taken together, Goffman and Foucault's perspectives on the historical development and nature of mental institutions demonstrate how the discourse of safety served to perpetuate the structure and systems of institutionalization. Stigma and fear operated as the primary forces behind the drive for safety from madness and rationalization for confinement. Practices within these spaces of confinement were legitimized by the same discourse of safety for the individual, the staff and the public from whom the inmates were securely removed.

## 3 Current Perspectives on Safety in Psychiatric Care

Deinstitutionalization, beginning in the 1950s in the United States and Canada, marked a new era in which total and long‐term sequestering of individuals within institutions was deemed unethical and asylums were closed. While historically, nursing ethics primarily referred to individual nurses’ personal characteristics including etiquette and manner, the development of professional ethics governing nursing practice shifted the principles informing treatment and care of patients and populations, including those with mental illness (Kangasniemi, Pakkanen, & Korhonen, 2015). In keeping with emergent mental health public policy and nursing professional ethics, the articulated aims of deinstitutionalization included returning individuals to home communities to restore freedom and autonomy (Hudson, 2016; Mezzina, 2014), and reducing or eliminating nursing practices grounded in punishment that were being societally reconceputalized as harmful (Gooding, 2016). Yet while the advancement of health care ethics has minimized the use of overt punishment in mental health care settings, numerous risk management strategies from the era of institutionalization continue to be utilized by nurses, including containment (i.e., locking doors to hospital units) and seclusion. The safety discourse as developed in the era of institutionalization continues to inform nursing practice, perpetuating and legitimizing these risk management strategies. Loukidou et al. (2010) argue that despite deinstitutionalization, mental health nursing as a profession remains institutionalized, in that the nature of mental health nursing practice borrows and extends directly from the care practices of institutions. The framework of deinstitutionalized care and the articulated shift towards safe and ethical health care provision for individuals with mental illness, although important and necessary advances in mental health care, obscure the harmful and unethical nature of risk management strategies utilized in inpatient psychiatric settings today.

As in the era of institutionalization, many nurses working in mental health care continue to hold the stigmatizing view that individuals with mental illness are dangerous and subsequently experience fear working in the inpatient setting (Johansson, Skärsäter, & Danielson, 2013; Linden & Kavanagh, 2012). Specifically, nurses fear unknown patients; those who are not familiar to the nurses from previous hospitalizations are deemed unpredictable and therefore unsafe (Camuccio, Chambers, Välimäki, Farro, & Zanotti, 2012; Johansson et al., 2013). Nurses’ fear of patient aggression increases the use of seclusion (De Benedictis et al., 2011), reduces therapeutic engagement (Johansson et al., 2013) and gives rise to unnecessary restrictions of patient autonomy such as cancelling off‐ground privileges (Doyal et al., 2009). The margin of acceptable behaviour remains narrow, with the inpatient environment characterized by boundaries and rules experienced as arbitrary by patients and by the nurses who are enforcing them, yet upheld through fear, stigma and the aim to ensure safety (Shattell et al., 2008; Vatne & Fagermoen, 2007). Although most unethical practices from the era of institutionalization have been identified as inhumane and discontinued, many of today's practices still resemble those from the past, including confinement from the outside world, seclusion and restraint, observation and surveillance, denial of leave and removal of personal belongings including clothes. The safety discourse, grounded in fear of individuals with mental illness, continues to legitimize the use of these practices in the same manner in which inhumane interventions were justified in the era of institutionalization.

The concept of the therapeutic relationship, as developed by Peplau (1952/1991), centres positive interpersonal interaction between nurse and client, with the client's needs and goals as the focus of the relationship. The therapeutic relationship has been integrated as a fundamental tenet of mental health nursing, which may suggest a dramatic shift in the treatment of individuals with mental illness and their experience of hospitalization in modern health care settings. Yet, the upholding of safety as the “highest aim” of mental health nursing (e.g., see Delaney & Johnson, 2008) may contradict the therapeutic relationship. Additionally, the notion that risk management strategies constitute treatment is perpetuated by modern care practices, and further displaces the centrality of the therapeutic relationship in care. For example, recent studies report that many nurses perceive of seclusion as an essential aspect of patient care (see Happell & Koehn, 2010; Landeweer et al., 2011). Similarly, Larsen and Terkelsen (2014) observed that nurses viewed “use of house rules and seclusion as important treatment activities rather than an oppressive practice” (p. 433). With safety as the primary value of inpatient care, nurses view risk management interventions designed to uphold safety as effective and beneficent treatment (e.g., Cutcliffe & Stevenson, 2008). Paterson et al. (2013) argue that to shift these “corrupt cultures” in which harmful interventions are misused and viewed as therapeutic, restraint must be reframed as treatment failure. The framing of risk management strategies as constituting treatment not only serves to legitimize harmful practices, but also obscures genuine treatment and interrelationships as envisioned by Peplau.

## 4 Risk Management: Nurse and Patient Perspectives

The current framework of safety in mental health nursing is founded in persistent stigmatizing beliefs of individuals with mental illness and continues to uphold institutionalization‐era practices of risk management that preclude the articulated aims of deinstitutionalized treatment. However, for direct care nurses, risk management strategies engender contradictory experiences of moral distress: Larsen and Terkelsen (2014) describe nurses’ experiences of distress both when utilizing containment interventions such as seclusion, articulating concerns that the methods are dehumanizing, and when *not* utilizing the interventions, citing safety concerns and the belief that treatment is being denied. Happell and Koehn (2010) report a concerning cognitive dissonance in which 87% of nurses regretted using seclusion yet almost half believed that patients felt safe and relieved after being secluded. The environment can also contribute to nurses’ dilemmas in treatment: in a comparative study of units with locked versus unlocked doors, nurses on unlocked units expressed anxiety about patients leaving the unit and harming themselves or others, while nurses on locked units were concerned that patient conflict and “disturbed behaviours” would increase (Gerace et al., 2015). In addition to feeling caught between interventions, nurses endorse moral distress surrounding the perceived loss of the therapeutic relationship as the foundation of psychiatric treatment (Austin, Bergum, & Goldberg, 2003). Nurses recognize that a care context in which safety concerns give rise to interventions steeped in control precludes opportunities for interpersonal engagement (Stevenson, Jack, O'Mara, & LeGris, 2015). Nurses articulate feeling powerless and beholden to a system which necessitates a certain type of mental health care, with few alternative options for care provision (Austin et al., 2003; Larsen & Terkelsen, 2014; VanDerNagel, Tuts, Hoekstra, & Noorthoorn, 2009). As nurses are the direct care providers and therefore engage in practices intended to maintain safety in the inpatient setting, the moral distress consistently articulated by nurses who utilize (and refrain from utilizing) these practices demonstrates the need for a re‐evaluation of the centrality of risk management in mental health nursing care.

In addition to contributing to nurses’ moral distress, risk management practices are experienced by patients as dehumanizing and traumatizing. Patients describe seclusion as humiliating and causing distress and fear (Kontio et al., 2012). The physical environments of psychiatric units are experienced as representative of the culture of care: units are perceived as jail‐like (Shattell et al., 2008), with locked doors representing exclusion from the outside world (Muir‐Cochrane et al., 2012). In Breeze and Repper's (1998) qualitative study of the experiences of patients labelled by health professionals as “difficult” in inpatient care, participants articulated their treatment as demonstrative of nurses’ power and control: examples include forced medications, denial of passes, restriction of participation in care and not being trusted by health care teams. Patients report experiencing fear in this environment, yet do not believe that nurses’ safety measures are effective for addressing risks (Stenhouse, 2013). Patient perspectives demonstrating harm further reinforce the understanding that while risk management strategies may be legitimized within current health care environments, these practices are unethical, both undermining patient autonomy and causing harm. Patient and nurse perspectives demonstrate that the framing of safety as the highest value in mental health nursing is not contributing to increased perceptions of safety, but rather is causing moral distress for direct care nurses and traumatizing patients.

## 5 Risk Exemplars

A safety lens in mental health nursing involves continuous assessment and management of potential and actual risks, through the use of established interventions supported by the organizational structure of the inpatient care environment. In this section, we provide four exemplars of identified risks and associated interventions, which demonstrate that risk management strategies utilized in the psychiatric inpatient setting are ineffective and harmful, and neither successfully create safe environments nor contribute to meaningful treatment.

### 5.1 Risk to self: suicide and constant observations

Suicide risk assessment and prevention are a critical component of upholding patient safety. Research on suicide risk focuses predominantly on identifying demographic factors associated with increased risk, such as younger age, living alone or unemployment (Bowers et al., 2010a; Stewart, Ross, Watson, James, & Bowers, 2013), behavioural and contextual risks including spending time in private areas of the unit (Bowers, Dack, Gul, Thomas, & James, 2011), or leaving the unit on passes when experiencing suicidal ideation (De Santis et al., 2015). However, nurses demonstrate a very low consistency in predicting suicide risk in hypothetical scenarios, suggesting that a risks‐based model based on demographic and behavioural factors is insufficient for preventing suicide (Paterson et al., 2008). In absence of clear risk identifiers, organizations utilize observation as a risk management intervention, including increased overall vigilance, direct patient observation and monitoring, and electronic surveillance of the unit (Bowers et al., 2010a; De Santis et al., 2015; Stewart & Bowers, 2012). Observation of a patient may be intermittent (occurring at random or scheduled intervals of time) or constant, with a nurse or other health care provider continually monitoring the individual, including in private spaces. The discourse of safety drives the ongoing use of this intervention: safety provides ethical justification for constant observation (Bowers et al., 2010a; Holyoake, 2013) and upholding safety is viewed as providing support and treatment for suicidal patients (Cutcliffe & Stevenson, 2008).

Nurses perceive constant observation as the safest intervention and endorse its efficacy in preventing inpatient suicide (De Santis et al., 2015; Holyoake, 2013). Despite the strong support for the use of constant observation and its primacy as a risk management strategy for inpatient suicide, research on this intervention has not successfully demonstrated its efficacy (Muralidharan & Fenton, 2012; Stewart et al., 2013). Bowers et al. (2011) describe multiple methods of completed suicides utilized by individuals on constant observation and suggest that nurses’ belief in the efficacy of the intervention contributes to reduced engagement and vigilance. Cutcliffe and Stevenson (2008) describe the use of constant observation as a “defensive and custodial practice” (p. 943) that has become synonymous with care provision, yet limits the provision of other forms of treatment or support, serving only as a band‐aid solution. Furthermore, constant observation contributes to loss of privacy, disempowerment and the perception of incarceration (Cox, Hayter, & Ruane, 2010). This practice, though widespread in its use, is unsupported by a substantive evidence base demonstrating efficacy in preventing suicides and can be conceived of as unethical in its harmful impact on the patients it is intended to protect.

### 5.2 Risk to others: inpatient violence and seclusion

The belief that individuals with mental illness are violent, unpredictable and dangerous is a pervasive stigmatizing view (Camuccio et al., 2012; Linden & Kavanagh, 2012), which has been shown to negatively affect nurses’ perceptions of personal safety (Bowers, Allan, Simpson, Jones, & van der Merwe, 2009). Patient seclusion in locked rooms as a violence risk management strategy is widespread, serving as a risk prevention and containment intervention (Landeweer et al., 2011). Despite attempts to reduce the use of this intervention internationally, one in five inpatients are reportedly secluded at least once in the duration of their hospitalization (Bullock, McKenna, Kelly, Furness, & Tacey, 2014).

The identification of demographic and diagnostic risk factors for aggression has been used extensively in research aimed at risk assessment and violence prevention (e.g., Daffern et al., 2010; Stewart & Bowers, 2013; Vruwink et al., 2012; Williamson et al., 2013). However, these risk factors are evaluated within a narrow context of searching for risk within individuals and research that evaluates the causes of inpatient violence more broadly identifies weak or absent associations with patient‐specific factors (Bowers et al., 2010b). When evaluated holistically, the primary cause of violence towards nurses in the inpatient setting appears to be patient–staff conflict (Kelly, Subica, Fulginiti, Brekke, & Novaco, 2015).

Seclusion in inpatient care settings is articulated by nurses as an intervention utilized in direct response to patient violence for the safety and protection of other patients and staff (Happell & Koehn, 2010; Zuzelo, Curran, & Zeserman, 2012). However, Bowers et al. (2010b) identified that the triggers for seclusion use in the clinical setting are primarily associated with non‐violent behaviours such as medication refusal, lack of rule following and absconding from the unit. Nursing behaviour is also a significant factor in seclusion room use with increased staff aggression towards patients correlated with increased seclusion use (Björkdahl, Hansebo, & Palmstierna, 2013; De Benedictis et al., 2011). Use of seclusion stems from and supports a “philosophy of physical separation” (Bowers et al., 2010b, p. 238), a culture in which this practice is legitimized and encouraged to promote safety (Landeweer et al., 2011; Paterson et al., 2013). Bowers et al. (2010b) demonstrate a strong correlation between the availability and use of this intervention, and argue that removal of seclusion rooms would not jeopardize safety or increase risks within the inpatient setting. While the practice of seclusion is legitimized through the aim of protecting nurses and other patients, Doyal et al. (2009) conclude that the line between necessity and convenience is frequently blurred and that seclusion is often utilized outside of its construction as a “necessary” intervention for upholding safety.

### 5.3 Risk to the public: absconding and door locking

Historically, the belief that individuals with mental illness pose a risk to the public has served as justification for confinement in jails and mental institutions. In today's inpatient psychiatric settings, patients who abscond from the unit continue to be viewed as potential risks to public safety (Gerace et al., 2015; van der Merwe, Bowers, Jones, Simpson, & Haglund, 2009). Muir‐Cochrane and Mosel (2008) identify absconding, with subsequent risk for violence and aggression towards the public, as a “major public health concern” (p. 373). While some minor measures, such as sign‐in/sign‐out books and careful breaking of bad news (such as a new diagnosis), are utilized to reduce absconding (Bowers, Simpson, & Alexander, 2005), the predominant risk management strategy is door locking, the environmental containment practice of continuously or intermittently locking the doors to the unit. Nurses view locked doors as protection for the public (van der Merwe et al., 2009) and perceive this intervention as facilitating control of the patient population and promoting security and safety (Johansson et al., 2013). On units with intermittent rather than continuous door locking, nurses describe utilizing this intervention during staffing shortages in an attempt to increase control of the population to uphold safety (van der Merwe et al., 2009). In a further demonstration of the perceived need to protect the public and the efficacy of door locking towards this aim, the state of Queensland in Australia has recently initiated continuous door locking across all adult mental health inpatient units (Grotto et al., 2014).

The contrast between the perceived benefits and demonstrated efficacy of this intervention is among the starkest within modern psychiatric care. Multiple research studies report no evidence that door locking reduces absconding, with patients frequently finding other methods of leaving the unit including by force, through following a visitor or staff member, or finding another exit from the unit (van der Merwe et al., 2009; Muir‐Cochrane & Mosel, 2008; Nijman et al., 2011). Yet the perception of door locking as effective remains so prevalent that the use of technology to complement and enhance door locking is emerging (Hearn, 2013; Nijman et al., 2011). While arguments persist for the use of these technologies as deterrents to absconding, the rates of absconding are unaffected by door security innovations (Nijman et al., 2011).

The hyperfocus on risk management and prevention obscures the complexity of causes of absconding from psychiatric inpatient units: rates of absconding are significantly higher on units with poor environments, including structural factors and increased verbal aggression (Nijman et al., 2011). Contextual reasons for absconding include fear, boredom, lack of privacy and concerns surrounding responsibilities at home (Muir‐Cochrane & Mosel, 2008), which door locking does not address. Units with locked doors demonstrate increases in patient anger and aggression as well as higher rates of seclusion use (Ashmore, 2008; Bowers et al., 2009; Muir‐Cochrane et al., 2012). Patients perceive the locking of unit doors as reducing autonomy and freedom (Ashmore, 2008), and experience increased shame, depression, powerlessness, isolation and exclusion (Muir‐Cochrane et al., 2012). Patients have also reported that the environment symbolizes restriction and control, and creates barriers to safe and effective treatment, including therapeutic engagement with nursing staff (Shattell et al., 2008). While door locking continues to be upheld as a necessary safety measure for protecting the public, the practice is ineffective and contributes to dehumanizing and indeed less safe care environments.

### 5.4 Risk to professional responsibility: blame and defensive practice

Crowe and Carlyle (2003) argue that due to the conceptualization of individuals with mental illness as inherently posing risks to self, others and the public, clinicians may be held directly responsible and blamed for emergent threats to safety. Minimal research has directly explored mental health nurses’ perceptions of their responsibilities for upholding safety in the clinical setting, or the impact of these perceptions on patient care. However, available literature on nurses’ perceptions of their responsibilities for managing risk in the inpatient environment provides insight into their continued use of risk management strategies as a means of protecting against blame. Nurses experience fear of adverse outcomes not only out of care for their patients, but also out of fear of blame: for example, nurses whose patients abscond report fear of punitive repercussions for a lapse in appropriate risk management (Gerace et al., 2015; Muir‐Cochrane et al., 2012). Nurses also report fear of litigation if their patients self‐harm or attempt suicide on the unit (Cutcliffe & Stevenson, 2008; De Santis et al., 2015). While negligent or irresponsible practice must not be accepted or ignored, adverse events occurring within the inpatient setting may nevertheless result in blame, sanction or litigation of the responsible nurse. Delaney and Johnson (2008) describe the role of inpatient mental health nurses as “hold[ing] 24‐hour accountability for the integrity of the inpatient environment” (p. 386), tasked with maintaining safety at all times. This role creates an impossible balance for nurses in the provision of patient care: in Manuel and Crowe's (2014) qualitative exploration of mental health nurses’ perceptions of clinical responsibility, nurses described the difficulty of weighing a patient's therapeutic needs against the pervasive “potential for blame in the organizational culture of risk management” (p. 388). Nurses revealed that while they desire to provide therapeutic care, this aim is overshadowed by the mandate to continually intervene to minimize risk and mitigate harm and to extensively document clear rationale for each clinical intervention to avoid blame.

Patients thus pose a risk to the upholding of safety itself, and to the professional responsibility of the nurses tasked with reducing risks. Fear of blame experienced by nurses in the care of clients in the psychiatric inpatient setting results in a defensive, rather than therapeutic, practice. Nurses undertaking constant observation of high‐risk patients describe following safety procedures and protocols precisely, not in order to provide optimal care, but to protect themselves against legal action in the event of an adverse outcome (MacKay, Paterson, & Cassells, 2005). Cutcliffe and Stevenson (2008) argue that close observation is in itself a defensive practice, which serves only to maintain physical safety of the patient and protect the nurse from litigation, as opposed to promoting therapeutic engagement or addressing underlying suicidality. Defensive practice serves as a risk management strategy for the risks patients pose to nurses’ responsibility for maintaining safety, although the very nature of this practice detracts from therapeutic engagement and meaningful treatment.

## 6 Shifting the Safety Discourse

These four exemplars illustrate the mechanisms through which the safety discourse operates to promote and legitimize nurses’ use of ineffective strategies for identifying and mitigating risks in mental health clinical settings. Despite harms experienced by patients ostensibly protected by these interventions, including traumatic and dehumanizing experiences and the perpetuation of restrictive and controlling environments, safety remains the primary aim of inpatient treatment. Goffman and Foucault's works demonstrate the historical context in which safety has legitimized and perpetuated harmful practices within psychiatric institutions; in modern nursing care, risk management strategies continue to create harms despite deinstitutionalization initiatives and the development of ethical standards for nursing practice. While safety must remain an important component of mental health nursing, truly supporting and empowering patients within the hospital setting involves discontinuing invasive and harmful practices legitimized through the safety discourse as articulated and operationalized in current nursing practice. To change the conceptualization and management of risk in psychiatric inpatient care, the concept of safety itself must be reframed, and other care practices and frameworks prioritized. We suggest two strategies for shifting the safety discourse within mental health nursing: re‐evaluating risk and shifting responsibility.

### 6.1 Re‐evaluating risk

Nursing care of patients in the psychiatric inpatient setting is fundamentally grounded in risk aversion. A risk averse lens of practice supports a focus on identification of risks in order to continuously implement prevention strategies. However, prediction of risk at the level of the individual patient is frequently inaccurate (Mulder, 2011), and at the population level, demographic and diagnostic factors are not predictive (Bowers et al., 2010b). The continued use of patient factors for prediction of risk promotes stereotyping and inappropriate use of interventions (Bullock et al., 2014). Risk management strategies are often misapplied and utilized primarily to uphold safety rather than support wellness. The safety discourse in inpatient psychiatric care is totalizing: risks are viewed as pervasive and absolute, with each potential risk factor requiring immediate management at the cost of meaningful treatment. A new conceptualization of safety must not only involve acknowledging the possibility of risk, but also seek to balance the value of safety with that of therapeutic relationships.

Dziopa and Ahern (2008) argue that to support effective individualized care of clients, nursing practices grounded in risk aversion must shift to a model of treatment flexibility. The authors state that nurses must have “the ability to interpret unit rules and evaluate the risks associated with bending them” (pp. 3–4). However, Collins (2012) notes that nurses may rule bend for reasons such as saving time or avoiding difficult tasks, actions which may ignore or introduce risks and jeopardize patient safety. An alternative to these uncritical approaches to rule bending is Hutchison's (1990) responsible subversion: rule bending in the context of comprehensively evaluating the situation and predicting potential outcomes, including risk, based on nursing knowledge and experience. Taking up responsible subversion in the psychiatric inpatient setting creates an alternative safety culture in which relative risks are critically evaluated in an iterative process, acknowledged appropriately when present and mitigated thoughtfully. An example of responsible subversion is explored by Gutridge (2010), in a “harm‐minimization” approach to self‐harm. Gutridge presents an ethical inquiry of health care providers’ responsibilities relating to patient self‐harm in the psychiatric inpatient setting and suggests an approach in which health care providers acknowledge that some self‐injury may occur on an individual's trajectory towards wellness. Gutridge suggests an alternative approach to nursing care of those at risk of self‐injury in which severity of self‐injurious behaviour and the potential for secondary risks, such as infection, are prioritized over striving to prevent *all* self‐injury. This approach of responsible subversion demonstrates that reintroduction of risk can be ethical when perceptions of risk are shifted. When risk is viewed as absolute within organizational structures, nurses who permit risk to emerge through failing to intervene appropriately are blamed. However, viewing risk as relative, and re‐introducing the possibility of risk into the clinical setting with harm minimization strategies and a therapeutic goal in mind, reduces practices grounded in fear of adverse events and provides nurses opportunities to provide meaningful treatment.

Re‐evaluating risk involves not only introducing flexibility of risk management, but also reconceptualizing values in care. The use of risk management strategies to uphold safety in the clinical setting demonstrates that these practices preclude nurses’ therapeutic engagement with patients. To promote meaningful and effective treatment, nurses must ground their practice in the foundation of the therapeutic relationship. The therapeutic relationship serves to centre empathy, listening and time spent in direct interaction, as primary components in treatment (McAndrew, Chambers, Nolan, Thomas, & Watts, 2014). Cutcliffe and Stevenson (2008) echo this view, advocating for reframing “talking as the centerpiece” (p. 943) for nursing care, arguing that engagement over containment strategies holds the power to support wellness. However, in mental health settings in which risks for self‐harm, violence and absconding pose genuine threats, neither must the therapeutic relationship overshadow nurses’ attention to the realities of risks. In Chiovitti's (2008) research on nursing care in psychiatric settings, nurse participants generated a theory of “protective empowering” in which safety is not afforded a hierarchical position, but remains a crucial component of care alongside therapeutic engagement and advocacy. Within this theory, safety does not imply the use of risk management strategies; rather, the concept is framed as protection through helping an individual meet their needs: this may include reassurance, assisting with self‐care, or providing information and choices. While safety remains a crucial value across inpatient environments, it must not eclipse other values or serve as the singular purpose of the psychiatric care. Centring the therapeutic relationship in nursing care provision supports recognition of clients’ true needs, which may include protection from risks, and empowers nurses and patients in addressing them.

### 6.2 Shifting responsibility

In psychiatric inpatient environments, nurses report anxiety in carrying the burden of responsibility for patient safety and utilize defensive rather than therapeutic practices in patient care to avoid blame or litigation. Organizational shifts are needed to support shared responsibility for upholding safety within the inpatient environment. The risk aversion mentality contributes to rigid and controlling environments, with inflexible rules and processes. While patients and nurses currently view rules as restrictive and arbitrary (Shattell et al., 2008), the effective development and use of unit guidelines can provide consistency and predictability (Isobel, 2015). To promote a shared commitment to a safe environment, the Safewards model for reducing conflict and containment advocates for nurses and patients developing unit guidelines collectively with a focus on mutual expectations (Bowers et al., 2015). These guidelines are posted publicly on units in order to uphold the collective nature of the space and shared responsibility for its environment and processes. Addressing safety through shared commitments shifts the framing of safety in the inpatient environment away from the model of sole nursing responsibility, a lens which legitimizes paternalistic practices.

Re‐centring responsibility for safety as shared between health care providers and clients involves changing perspectives on where risk is situated—from the individual to the health care context. To shift responsibility, risk must be relocated. For example, Sun, Long, Boore, and Tsao (2006) state that nursing care of an individual at risk for suicide includes “protecting patients from dangerous items” (p. 684), a framing that locates the risk in the environment, as opposed to within the patient. Similarly, the Safewards model suggests that health care providers actively identify the potential for a patient receiving bad news and develop interventions for discussing and debriefing this news (Bowers et al., 2015). This intervention likewise relocates risk, suggesting that the event of receiving bad news is itself the source of risk in its potential for negatively impacting a patient's emotional safety. These relocations of risk align with that of hospital environments outside of psychiatry, and create new possibilities for integrating patient safety, in its conceptualization as protection from iatrogenic harms, into psychiatric care.

When risk is located in the individual, a process read through stigmatizing beliefs surrounding mental illness, patients are held responsible and therefore blamed for adverse events. Warner (2010) argues that internalized stigma experienced by individuals with mental illness directly contributes to self‐blame and thus to dependency on others for treatment and support. The recovery model of mental health care seeks to disentangle the concepts of risk and blame, with clients assuming responsibility for actions taken towards wellness, though not blame for symptoms or illness (McKenna et al., 2014). In this model, nurses support clients in taking responsibility and accountability for treatment without abdicating their own professional responsibility for protection (Manuel & Crowe, 2014). Due to the model's focus on community re‐integration and development of meaning in life, recovery‐oriented mental health care initiatives and research into the efficacy of the model have predominantly targeted community nursing settings (Kidd et al., 2014). However, within the inpatient setting, nurses can adopt recovery‐oriented approaches to support clients in increasing responsibility for self‐management of medications and symptoms, and empower clients in peer support and teaching (Leamy, Bird, Le Boutillier, Williams, & Slade, 2011). These actions support personal responsibility through empowerment while avoiding blame for potential risk associated with mental illness symptoms.

Gutridge (2010) states, “development of judgement and self‐worth [is] being afforded the freedom to act” (p. 90), yet our current safety frameworks preclude this freedom. A shift in autonomy and responsibility for care is needed not only for reducing blame placed on those we are purporting to treat, but also for supporting autonomy itself as a therapeutic intervention. While mandated treatment poses a challenge to nurses promoting autonomy in the inpatient setting, all nurses can utilize a strengths‐based approach in patient care and centre freedom of choice (McKeown, Jones, Wright, Paxton, & Blackmon, 2016). Doyal et al. (2009) state that all individuals, regardless of their involuntary status or level of insight, retain “residual autonomy” which in nursing care of clients should serve “as the foundation on which to help patients regain their full competence” (p. 508).

## 7 Conclusions

For nurses working within mental health inpatient care settings, the safety discourse frames the nature of care provision, informing identification of risks posed by the clients in their care and the interventions utilized to manage these risks. Safety is articulated as the paramount aim of inpatient psychiatric care, yet this seemingly beneficent value is rooted in fear, stigma, and a history of institutionalization. Nursing practices aimed to uphold safety in inpatient settings are ineffective and harmful to both patients and nurses, yet their continual use is legitimized by the articulation and operationalization of the safety value. While safety is a crucial component of inpatient psychiatric nursing care, its framing and use must shift in order to create environments perceived as truly safe and to support meaningful therapeutic engagement and treatment.

## Note

1 Foucault demonstrates that mental illness, the medicalization of madness, is a relatively recent construct. Although madness is not a socially accepted contemporary term, it is utilized here to avoid anachronistic language in relation to Foucault's text.
